# Auditory-Somatosensory Temporal Sensitivity Improves When the Somatosensory Event Is Caused by Voluntary Body Movement

**DOI:** 10.3389/fnint.2016.00042

**Published:** 2016-12-16

**Authors:** Norimichi Kitagawa, Masaharu Kato, Makio Kashino

**Affiliations:** ^1^NTT Communication Science Laboratories, NTT CorporationKanagawa, Japan; ^2^Center for Baby Science, Doshisha UniversityKyoto, Japan; ^3^School of Engineering, Tokyo Institute of TechnologyKanagawa, Japan

**Keywords:** voluntary action, temporal sensitivity, multisensory integration, involuntary action, auditory perception, somatosensory perception

## Abstract

When we actively interact with the environment, it is crucial that we perceive a precise temporal relationship between our own actions and sensory effects to guide our body movements. Thus, we hypothesized that voluntary movements improve perceptual sensitivity to the temporal disparity between auditory and movement-related somatosensory events compared to when they are delivered passively to sensory receptors. In the voluntary condition, participants voluntarily tapped a button, and a noise burst was presented at various onset asynchronies relative to the button press. The participants made either “sound-first” or “touch-first” responses. We found that the performance of temporal order judgment (TOJ) in the voluntary condition (as indexed by the just noticeable difference (JND)) was significantly better (*M* = 42.5 ms ± 3.8 SEM) than that when their finger was passively stimulated (passive condition: *M* = 66.8 ms ± 6.3 SEM). We further examined whether the performance improvement with voluntary action can be attributed to the prediction of the timing of the stimulation from sensory cues (sensory-based prediction), kinesthetic cues contained in voluntary action, and/or to the prediction of stimulation timing from the efference copy of the motor command (motor-based prediction). When three noise bursts were presented before the target burst with regular intervals (predictable condition) and when the participant’s finger was moved passively to press the button (involuntary condition), the TOJ performance was not improved from that in the passive condition. These results suggest that the improvement in sensitivity to temporal disparity between somatosensory and auditory events caused by the voluntary action cannot be attributed to sensory-based prediction and kinesthetic cues. Rather, the prediction from the efference copy of the motor command would be crucial for improving the temporal sensitivity.

## Introduction

When we actively interact with the environment in everyday life, most of our body movements can generate impact sounds that provide fine temporal information regarding our body-environment interactions. Such auditory feedback of body movements can affect our motor behavior (e.g., Tajadura-Jiménez et al., 2016). For example, tapping behavior synchronized to a regular auditory sequence can be distracted by a subtle temporal irregularity inserted into the sequence (Repp, 2000; Kato and Konishi, 2006). To achieve precise control of body movements, our sensorimotor system has to assess the fine temporal relationship among voluntary actions and sensory feedback from multiple sensory modalities. Thus, our perceptual system would be more sensitive to temporal disparity between events in different sensory modalities when we voluntarily cause those sensory events compared to when the sensory events are delivered to our sensory receptors passively. We hypothesized that voluntary movements improve perceptual sensitivity to temporal disparity between auditory and movement-related somatosensory events in cases where there is a strong causal relationship between voluntary movements and those sensory events.

Three possible components contained in voluntary movements may improve multisensory temporal sensitivity compared to temporal sensitivity without body movements. The first component concerns the predictability of the timing of sensory events. When we voluntarily make a body movement, such as making a finger tap on a surface, we can know when we move the finger and when we will receive sensory feedback from the finger tap and thus can pay attention to the moment the finger contacts the surface. Since attention to a particular moment in time can enhance temporal resolution between sensory events around the attended moment (Correa et al., 2006), the predictability of the timing of sensory events might improve multisensory temporal sensitivity. The second component involves kinesthetic cues contained in voluntary movements. During body movements, we receive kinesthetic feedback from the moving body part irrespective of whether the movement is voluntary or involuntary. Such online kinesthetic cues (as well as the prediction of the timing of sensory events from those kinesthetic cues) might be useful in assessing the temporal relationship between feedback from multiple sensory modalities. The third component is related to the existence of an efference copy of the motor command of voluntary movement. The efference copy of a motor command is used to predict sensory feedback that estimates the sensory consequences of voluntary movement (Wolpert and Ghahramani, 2000). This prediction of sensory feedback generated by the motor system (i.e., motor-based prediction) might improve multisensory temporal sensitivity.

A seminal study (Adelstein et al., 2003) provides possible support for our hypothesis that voluntary movements improve perceptual sensitivity to temporal disparity between multisensory events caused by the voluntary movement. They measured perceptual sensitivity to temporal delays of auditory events from voluntary strike impacts of a hammer on a block and reported that the just noticeable difference (JND), which represents the observers’ sensitivity to temporal asynchronies and represents the smallest temporal disparity observers can reliably notice (Keetels and Vroomen, 2012), was around 25 ms. This value of the JND seems rather small compared to those usually reported for passively delivered auditory-somatosensory stimuli (e.g., about 80 ms for non-trained participants reported by Zampini et al., 2005). However, since multisensory temporal sensitivity can be affected by several factors such as the temporal profile of stimuli (Ley et al., 2009), training in the task (Zampini et al., 2005), spatial disparity between stimuli and stimulus complexity (for a review see Keetels and Vroomen, 2012), directly comparing these magnitudes of JNDs obtained in the different experimental conditions is difficult. Also note that careful experimental control is necessary in order to disentangle the effects of the three components involved in voluntary movements on the possible improvement of temporal sensitivity reported by Adelstein et al. (2003).

More recent studies have tried to further assess the effects of voluntary movements on multisensory temporal perception by introducing an involuntary movement condition in which the participants’ arm or finger is moved passively by a device (Frissen et al., 2012; Nishi et al., 2014; Hao et al., 2015, 2016). They compared the effects of voluntary and involuntary movements on multisensory temporal sensitivity, but the findings are not consistent even among the studies from the same laboratory. Although no significant differences in JNDs for the temporal disparity of auditory-somatosensory events between the voluntary and involuntary movement conditions have been reported (Frissen et al., 2012; Hao et al., 2015), improvement (Nishi et al., 2014) and even deterioration (Hao et al., 2016) of the JNDs has also been observed in the voluntary movement condition compared to passive and involuntary ones. The reason for this inconsistency between the findings of the previous studies is unclear. However, a critical point concerning the present interest is that, in these studies, there was no causal relationship between the voluntary movements and the auditory-somatosensory events: the somatosensory events were not caused by the voluntary movements *per se*; instead, they were applied passively during body movement at a random timing relative to them. Therefore, the findings of these previous studies can provide some implications only for the temporal perception during voluntary movements of the body in general, as it has been reported that somatosensory events are perceived as displaced in space and time during voluntary body movements (Dassonville, 1995; Watanabe et al., 2009). The effects of voluntary movements on perceptual sensitivity to temporal disparities between multisensory events under a strong causal relationship between the voluntary movements and the multisensory events are still unknown, despite their importance for the understanding the nature of the temporal aspects of our active body-environment interactions.

The present study tried to dissociate the effects of the three components—sensory-based predictability of the timing of multisensory events, kinesthetic cues of body movements and motor-based prediction from the efference copy of a motor command—involved in voluntary movements that might improve multisensory temporal sensitivity (compared to that without body movements) when there is a strong causal relationship between the voluntary movements and the sensory events. In our experiment, we measured perceptual sensitivity to temporal disparity between movement-related somatosensory and auditory events in four conditions: *passive*, *predictable, involuntary* and *voluntary* conditions. Table 1 shows the availability of the three components in the four experimental conditions. In the passive condition, participants’ static finger was tapped passively and none of the three components were available. In the predictable condition, the participants’ finger was tapped passively and a sensory cue was provided that enabled the participants to make a sensory-based prediction of the timing of the sensory events. In the involuntary condition, a device moved the participants’ finger to make it tap on a surface. This provided online kinesthetic cues for assessing the temporal relationship between the audiotactile events, and the kinesthetic cues could also be used to predict the event timing. In the voluntary condition, participants made a voluntary finger tap on a surface and all three components were available. In all conditions, a brief sound was presented at various stimulus onset asynchrony (SOA) relative to the somatosensory event. We employed a temporal order judgment (TOJ) task for assessing the temporal sensitivity (Vroomen and Keetels, 2010).

**Table 1:** **Availability of three components that might improve temporal sensitivity in the four experimental conditions**.

	Available component		
Experimental condition	Sensory-based prediction	Online kinesthetic cues	Motor-based prediction
Passive
Predictable	◯		
Involuntary	◯	◯	
Voluntary	◯	◯	◯

## Materials and Methods

### Participants

Fifteen right-handed participants (seven males and eight females, aged 21–35) took part in the experiments. They were naïve as to the purpose of the study. This study was carried out in accordance with the recommendations of ethical guidelines of NTT Communication Science Laboratories. All participants gave written informed consent in accordance with the Declaration of Helsinki. The protocol was approved by the ethical committee of NTT Communication Science Laboratories.

### Apparatus and Materials

**Figure 1:**
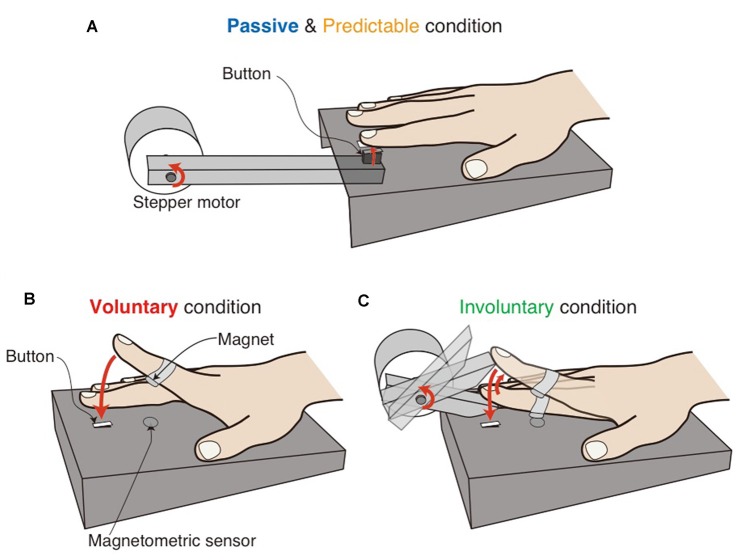
**Schematic illustrations of the experimental setups in the passive and predictable conditions (A)**, the voluntary condition **(B)** and the involuntary condition **(C)**. The participants’ hand and the setup were hidden from the participants’ view. In the voluntary and involuntary conditions **(B,C)**, a magnetometric sensor monitored the distance to a magnet attached to the finger, enabling us to present the sound before the button was touched.

The experiments were conducted in a sound-attenuated chamber. The participants sat at a table and rested their right forearm so that their right forefinger was located just above a button fixed in a box (Figure 1A). Their hand and the apparatus were hidden from their view. The experiment was controlled by MATLAB (MathWorks) on a computer and custom-made apparatus. The sound stimulus was a white-noise burst of 10-ms duration presented at 70 dB SPL through closed-ear headphones (Sennheiser HDA200). Two loudspeakers were used to present white noise at 80 dB SPL to completely mask any noise made by the apparatus and finger movements. The headphones attenuated the masking noise to 60 dB SPL. The somatosensory stimulus in the passive and predictable conditions was delivered to the right forefinger pad by a moving bar with the contactor of a micro-switch (5 mm × 15 mm; see Figure 1A). The voluntary (i.e., self-generated) somatosensory stimulus consisted of a voluntary finger tap to the same micro-switch (Figure 1B). The involuntary somatosensory stimulus consisted of an involuntary finger tap to the micro-switch. That is, the participants’ finger was lifted and then released by a moving bar to make the finger tap the button involuntarily (Figure 1C). A magnet was fixed at the middle of the finger, and a magnetometric sensor located inside the box monitored the distance to the finger (Figures 1B,C), allowing the sound stimulus to be presented prior to the voluntary and involuntary touch.

### Procedure

On each trial in the all conditions, the participants’ task was to make TOJs between the auditory and somatosensory events (“touch-first” or “sound-first”). They depressed foot pedals (one under the left foot and another under the right foot) throughout each block of trials. They raised their left (right) foot briefly to indicate a sound-first (touch-first) response. In the passive and predictable conditions, each trial started after a random interval (800–1800 ms) from the onset of light-emitting diode (LED) illumination. The bar then moved upwards to tap the participants’ right forefinger pad. In the voluntary condition, the participants started the trial with their forefinger lifted around 7 cm above the button, and they voluntarily tapped the button at a time of their choice after an LED was illuminated to indicate the start of the trial. In the involuntary condition, the participants’ finger was lifted around 7 cm above the button. After a random interval (800–1800 ms) from the onset of LED illumination, it was released by the moving bar to make the finger tap the button involuntarily. It took around 150 ms for the finger to hit the button in the voluntary and involuntary conditions.

**Figure 2:**
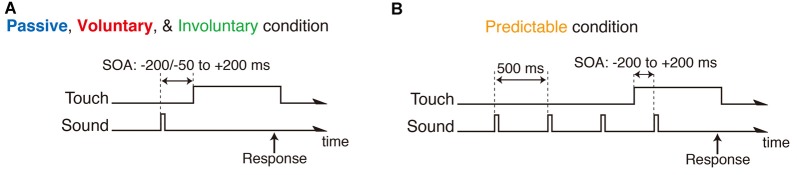
**Time course of trial in passive, voluntary and involuntary conditions (A)**, and in the predictable condition **(B)**. Negative stimulus onset asynchrony (SOA) indicates sound before touch.

The SOA between the auditory and somatosensory events was either ±200, ±150, ±100, ±80, ±60, ±40 or ±20 ms (negative value indicates sound before touch) in the passive and predictable conditions, whereas the SOA in the voluntary and involuntary conditions was either −50, −40, −30, −20, −15, −10, −5, 20, 40, 60, 80, 100, 150 or 200 ms (Figure 2). The minimum value of the SOA of −50 ms was determined by the maximum finger-sensor distance that could be measured reliably by the magnetometric sensor. The negative SOAs in the voluntary and involuntary conditions were measured as the actual time difference between the sound onset and the onset of touching the button. This was done because there was trial-to-trial variability in the actual SOAs due to slight differences in the finger movements, and then the SOAs were split into six bins of 10-ms width with the centers of −55, −45, −35, −25, −15, −5 ms for further analyses. In the predictable condition, a noise burst that was the same as the target noise burst was presented three times prior to the target burst at regular 500-ms intervals (Figure 2B) so that the participants could predict the target tone and pay attention to that moment. Each condition (passive, voluntary, predictable and involuntary) was separated into five experimental blocks in which each SOA was presented ten times in random order (in total 50 trials for each SOA). Twenty experimental blocks (4 conditions × 5 blocks) were presented in random order.

## Results

**Figure 3:**
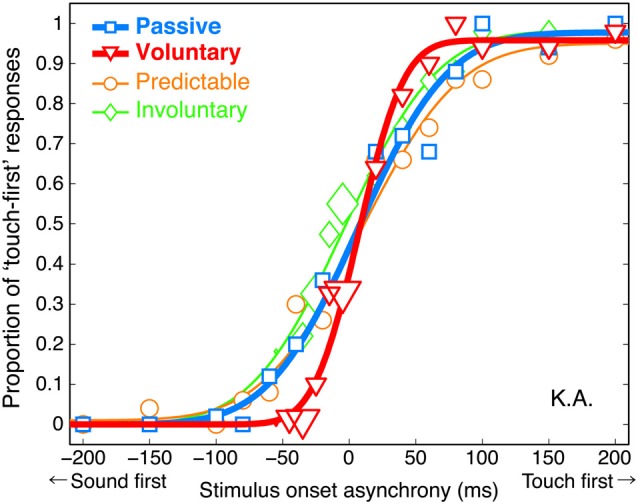
**Psychometric functions (proportion of “touch-first” responses as a function of SOA) from a typical participant, K.A.** The size of each plot reflects the number of trials. Each SOA was presented an average of 50 times.

We found that the voluntary action influenced the temporal sensitivity. The proportion of “touch-first” responses was plotted as a function of SOA (a negative value indicates sound before touch), and cumulative Gaussian functions were fitted to each participant’s data (Wichmann and Hill, 2001) for each condition. A typical participant performed the TOJ task almost perfectly at SOAs longer than 100 ms in the passive condition (Figure 3). In the voluntary condition, the performance was almost perfect even at SOAs as short as 50 ms, resulting in a steeper psychometric function in the voluntary condition than in the passive one. The psychometric functions in the involuntary and predictable conditions had slopes similar to that in the passive condition.

**Figure 4:**
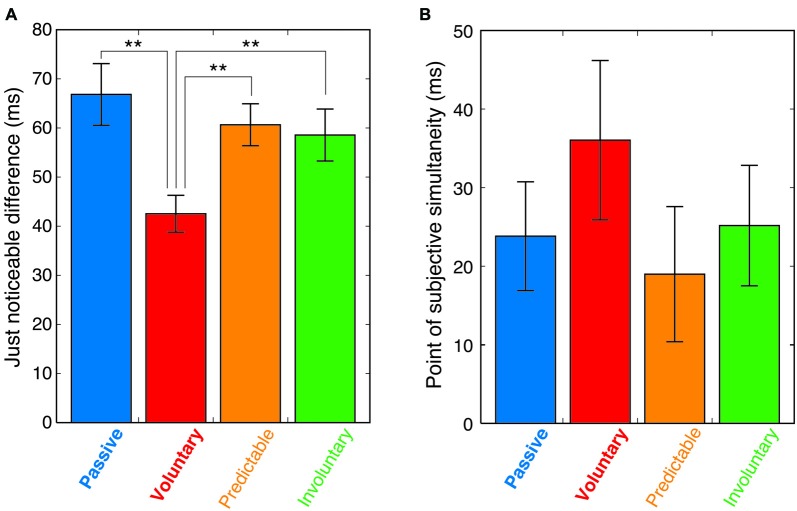
**(A)** Mean just noticeable differences (JNDs) in the four conditions. Error bars indicate the standard error of the mean. Asterisks show significance of difference (**p < 0.01). **(B)** Mean point of subjective simultaneity (PSS) in the four conditions with error bars of standard error of the mean.

We calculated the JND and the point of subjective simultaneity (PSS). The JND, which corresponds to the standard deviation of the fitted function, represents the observers’ sensitivity to temporal asynchronies. PSS is assumed that at this SOA, the information from the different modalities is perceived as being maximally simultaneous (Keetels and Vroomen, 2012). A repeated-measures analysis of variance of the JNDs revealed a significant main effect of condition (*F*_(3,42)_ = 10.03, *p* < 0.0001). *Post hoc* comparisons (Tukey’s honest significant difference) showed that the JND in the voluntary condition was significantly smaller than in the passive, predictable and involuntary conditions (*p* < 0.01), with no significant differences between those three conditions (Figure 4A). While the observed JNDs in the passive condition were comparable to those reported in previous studies (e.g., Zampini et al., 2005), the voluntary movement reduced the JND by 36% from that obtained in the passive condition (Figure 4A; the mean JND was 66.8 (±6.3 SEM) in the passive condition and 42.5 (±3.8 SEM) in the voluntary condition). The same analysis of the PSSs revealed no significant main effect of condition (*F*_(3,42)_ = 1.19, *p* = 0.32, Figure 4B).

## Discussion

Previous studies have examined the effects of voluntary movements on temporal perception of sensory events without causal relationship between voluntary movements and sensory events (Frissen et al., 2012; Nishi et al., 2014; Hao et al., 2015). In contrast, the present study examined whether voluntary movements improve perceptual sensitivity to temporal disparity between mutltisensory events when those events are virtually caused by the voluntary movements. We tried to determine which components involved in voluntary movements (the sensory-based prediction, kinesthetic cues and motor-based prediction) are responsible for the improvement in temporal sensitivity. We found that temporal sensitivity in the voluntary condition was significantly higher than in the passive condition. The lack of improvement in the temporal sensitivity in the predictable condition suggests that predicting the timing of sensory events from sensory cues (i.e., sensory-based prediction) does not improve the temporal sensitivity. As the temporal sensitivity in the involuntary condition was almost the same as that in the passive condition, kinesthetic cues contained in voluntary movements cannot explain the improvement of the temporal sensitivity. These results suggest that the efference copy of the motor command of voluntary movement plays an important role in improving the temporal sensitivity (see Table 1). The timing of sensory feedback predicted by the motor system (i.e., motor-based prediction) would improve perceptual temporal sensitivity between the sensory events caused by the voluntary movements.

Although no improvement in the temporal sensitivity was observed in the predictable condition, a previous study (Shi et al., 2008) reported that the presentation of visual motion trajectory could improve sensitivity to temporal disparity between a subsequent visual collision and auditory event. This suggests that sensory-based prediction from visual anticipatory cues can improve audiovisual temporal sensitivity to causally related sensory events (see also Van Eijk, 2008, Chapter 4 for similar results). In our predictable condition, the auditory sequence with 500-ms intervals was presented as an auditory anticipatory cue. The threshold for detecting irregularity such an auditory sequence would be around 20 ms (Halpern and Darwin, 1982), which is fairly smaller than the JNDs observed in the present study. Thus, the auditory sequence itself would contain sufficient anticipatory information about the stimulus timing for improving temporal sensitivity. In the involuntary condition, the continuous kinesthetic cue could also be used to predict the timing of the finger tap, but no improvement in temporal sensitivity was observed. Future studies should examine what kind of sensory predictive information can enhance multisensory temporal perception.

One may argue that the results can be explained by sensory attenuation caused by a voluntary movement (e.g., Blakemore et al., 1998). However, this argument is implausible in two respects. First, attenuated sensation would degrade TOJ performance (Terao et al., 2008), whereas voluntary movements improved the performance in the present study. Second, attenuated sensation might lead to slower latency of the sensation (e.g., Smith, 1933), which would result in a PSS shift rather than a JND change.

The PSSs observed in the passive condition were slightly biased to a positive value (i.e., the somatosensory events led the auditory event). This direction of bias in PSS has been reported repeatedly for passive audiotactile TOJ (Zampini et al., 2005; Nishi et al., 2014; Hao et al., 2015), suggesting that the tactile event should be presented slightly earlier than the auditory one for it to be perceived simultaneously with the auditory event. We observed no significant differences of PSSs between the conditions. However, it should be noted that the range of the SOAs in the voluntary and involuntary conditions (−50 to 200 ms) were different from those in the passive and predictable conditions (−200 to 200 ms). The absence of significant differences in the PSSs between the conditions might reflect centering bias (i.e., observers’ tendency to center their range of responses on the range of stimuli; Poulton, 1979), resulting from the different stimulus distributions between the conditions.

It has been reported that voluntary movements also enhance the action-effect binding process by attracting an action and its effect to each other in perceived time, with their tending to lower the perceptual temporal sensitivity (Haggard et al., 2002; Engbert et al., 2008; Wenke and Haggard, 2009). This “intentional binding” has typically been reported for pairs of voluntary movements and their effects with an onset asynchrony of 200–600 ms, which is obviously the supra-threshold for detecting temporal disparity. However, the present study revealed that perceptual temporal sensitivity improves within a 100-ms asynchrony range relative to the voluntary movement. We suggest that the central nervous system has at least two neural mechanisms that process the temporal relationship between voluntary movements and sensory effects. One improves the temporal resolution at the *perceptual* level within a time range immediately surrounding voluntary actions (less than 100 ms asynchrony), thus assisting precise motor control. The other binds voluntary movements and their effects that occur close together in time but clearly asynchronously (more than 200 ms up to several hundreds of milliseconds), thereby enhancing the causal relationship between actions and effects in *conscious awareness*. It would be interesting to examine the relationship between these mechanisms in future research.

In conclusion, we found that perceptual sensitivity to temporal disparity between auditory and somatosensory events can be higher when the sensory events are caused by voluntary body movements than when they are passively delivered. This improvement cannot be attributed to sensory-based prediction of the timing of multisensory events or to kinesthetic cues of body movements. Rather, motor-based prediction of the timing of sensory feedback from efference copy of motor command would improve temporal sensitivity. To best of our knowledge, this is the first empirical evidence that perceptual temporal sensitivity can be improved by motor-based prediction generated by the motor system. This process would provide an accurate temporal comparison of predicted and actual sensory effects, thus assisting precise temporal control of body movement in body-environment interactions. Several issues remain for future investigation. For instance, it is uncertain what is exactly improved by voluntary movement (e.g., timing encoding of the sensory events) and what differences between sensory-based and motor-based prediction improve the temporal sensitivity (e.g., difference in temporal precision of predictions, or any qualitative or functional differences of predictions). It would also be worth investigating neural mechanisms involved in the influence of motor-based prediction on improvement of perceptual temporal sensitivity.

## Author Contributions

All authors contributed to designing the study, running experiment, analyzing data, writing the manuscript.

## Conflict of Interest Statement

The authors declare that the research was conducted in the absence of any commercial or financial relationships that could be construed as a potential conflict of interest.

## References

[B1] Adelstein B. D. Begault D. R. Anderson M. R. Wenzel E. M. (2003). “Sensitivity to haptic-audio asynchrony,” in *Proceedings of the 5th International Conference on Multimodal Interfaces* (New York, NY), 73–76.

[B2] Blakemore S. J. Wolpert D. M. Frith C. D. (1998). Central cancellation of self-produced tickle sensation. Nat. Neurosci. 1, 635–640. 10.1038/2870 10196573

[B3] Correa A. Sanabria D. Spence C. Tudela P. Lupiáñez J. (2006). Selective temporal attention enhances the temporal resolution of visual perception: evidence from a temporal order judgment task. Brain Res. 1070, 202–205. 10.1016/j.brainres.2005.11.094 16403468

[B4] Dassonville P. (1995). Haptic localization and the internal representation of the hand in space. Exp. Brain Res. 106, 434–448. 10.1007/bf00231066 8983987

[B5] Engbert K. Wohlschläger A. Haggard P. (2008). Who is causing what? The sense of agency is relational and efferent-triggered. Cognition 107, 693–704. 10.1016/j.cognition.2007.07.021 17825813

[B6] Frissen I. Ziat M. Campion G. Hayward V. Guastavino C. (2012). The effects of voluntary movements on auditory–haptic and haptic–haptic temporal order judgments. Acta Psychol. 141, 140–148. 10.1016/j.actpsy.2012.07.010 22964054

[B7] Haggard P. Clark S. Kalogeras J. (2002). Voluntary action and conscious awareness. Nat. Neurosci. 5, 382–385. 10.1038/nn827 11896397

[B8] Halpern A. R. Darwin C. J. (1982). Duration discrimination in a series of rhythmic events. Percept. Psychophys. 31, 86–89. 10.3758/bf03206204 7070941

[B9] Hao Q. Ogata T. Ogawa K. Kwon J. Miyake Y. (2015). The simultaneous perception of auditory-tactile stimuli in voluntary movement. Front. Psychol. 6:1429. 10.3389/fpsyg.2015.01429 26441799 PMC4585164

[B10] Hao Q. Ora H. Ogawa K. Ogata T. Miyake Y. (2016). Voluntary movement affects simultaneous perception of auditory and tactile stimuli presented to a non-moving body part. Sci. Rep. 6:33336. 10.1038/srep33336 27622584 PMC5020736

[B11] Kato M. Konishi Y. (2006). Auditory dominance in the error correction process: a synchronized tapping study. Brain Res. 1084, 115–122. 10.1016/j.brainres.2006.02.019 16556436

[B12] Keetels M. Vroomen J. (2012). “Perception of synchrony between the senses,” in Neural Basis of Multisensory Processes, eds Murray M. M. Wallace M. T. (Boca Raton, FL: CRC Press), 147–177. 22593865

[B13] Ley I. Haggard P. Yarrow K. (2009). Optimal integration of auditory and vibrotactile information for judgments of temporal order. J. Exp. Psychol. Hum. Percept. Perform. 35, 1005–1019. 10.1037/a0015021 19653745

[B14] Nishi A. Yokoyama M. Ogawa K. Ogata T. Nozawa T. Miyake Y. (2014). Effects of voluntary movements on audio-tactile temporal order judgment. IEICE Trans. Inf. Syst. E97.D, 1567–1573. 10.1587/transinf.e97.d.1567

[B15] Poulton E. C. (1979). Models for biases in judging sensory magnitude. Psychol. Bull. 86, 777–803. 10.1037/0033-2909.86.4.777 482484

[B16] Repp B. H. (2000). Compensation for subliminal timing perturbations in perceptual-motor synchronization. Psychol. Res. 63, 106–128. 10.1007/pl00008170 10946585

[B17] Shi Z. Hirche S. Schneider W. X. Müller H. (2008). “Influence of visuomotor action on visual-haptic simultaneous perception: a psychophysical study,” in *2008 6th Symposium on Haptic Interfaces for Virtual Environment and Teleoperator Systems*, (Reno, NV), 65–70. 10.1109/haptics.2008.4479915

[B18] Smith W. F. (1933). The relative quickness of visual and auditory perception. J. Exp. Psychol. 16, 239–257. 10.1037/h0071379

[B19] Tajadura-Jiménez A. Marquardt T. Swapp D. Kitagawa N. Bianchi-Berthouze N. (2016). Action sounds modulate arm reaching movements. Front. Psychol. 7:1391. 10.3389/fpsyg.2016.01391 27695430 PMC5025518

[B20] Terao M. Watanabe J. Yagi A. Nishida S. (2008). Reduction of stimulus visibility compresses apparent time intervals. Nat. Neurosci. 11, 541–542. 10.1038/nn.2111 18408716

[B21] Van Eijk R. L. (2008). Audio-visual Synchrony Perception. Thesis, Netherlands: Technische Universiteit Eindhoven.

[B22] Vroomen J. Keetels M. (2010). Perception of intersensory synchrony: a tutorial review. Atten. Percept. Psychophys. 72, 871–884. 10.3758/APP.72.4.871 20436185

[B23] Watanabe J. Nakatani M. Ando H. Tachi S. (2009). Haptic localizations for onset and offset of vibro-tactile stimuli are dissociated. Exp. Brain Res. 193, 483–489. 10.1007/s00221-009-1711-y 19198817

[B24] Wenke D. Haggard P. (2009). How voluntary actions modulate time perception. Exp. Brain Res. 196, 311–318. 10.1007/s00221-009-1848-8 19471909 PMC2700248

[B25] Wichmann F. A. Hill N. J. (2001). The psychometric function: I. Fitting, sampling and goodness of fit. Percept. Psychophys. 63, 1293–1313. 10.3758/bf03194544 11800458

[B26] Wolpert D. M. Ghahramani Z. (2000). Computational principles of movement neuroscience. Nat. Neurosci. 3, 1212–1217. 10.1038/81497 11127840

[B27] Zampini M. Brown T. Shore D. I. Maravita A. Röder B. Spence C. (2005). Audiotactile temporal order judgments. Acta Psychol. 118, 277–291. 10.1016/j.actpsy.2004.10.017 15698825

